# GNIFdb: a neoantigen intrinsic feature database for glioma

**DOI:** 10.1093/database/baac004

**Published:** 2022-02-12

**Authors:** Wendong Li, Ting Sun, Muyang Li, Yufei He, Lin Li, Lu Wang, Haoyu Wang, Jing Li, Hao Wen, Yong Liu, Yifan Chen, Yubo Fan, Beibei Xin, Jing Zhang

**Affiliations:** Key Laboratory for Biomechanics and Mechanobiology of Ministry of Education, Beijing Advanced Innovation Centre for Biomedical Engineering, School of Engineering Medicine, School of Biological Science and Medical Engineering, Beihang University, No.37 Xueyuan Road, Haidian District, Beijing 100083, P. R. China; Key Laboratory for Biomechanics and Mechanobiology of Ministry of Education, Beijing Advanced Innovation Centre for Biomedical Engineering, School of Engineering Medicine, School of Biological Science and Medical Engineering, Beihang University, No.37 Xueyuan Road, Haidian District, Beijing 100083, P. R. China; Department of Plant Genetics and Breeding, State Key Laboratory of Plant Physiology and Biochemistry & National Maize Improvement Center, China Agricultural University, No.17 Qinghua East Road, Haidian District, Beijing 100193, P. R. China; Key Laboratory for Biomechanics and Mechanobiology of Ministry of Education, Beijing Advanced Innovation Centre for Biomedical Engineering, School of Engineering Medicine, School of Biological Science and Medical Engineering, Beihang University, No.37 Xueyuan Road, Haidian District, Beijing 100083, P. R. China; Key Laboratory for Biomechanics and Mechanobiology of Ministry of Education, Beijing Advanced Innovation Centre for Biomedical Engineering, School of Engineering Medicine, School of Biological Science and Medical Engineering, Beihang University, No.37 Xueyuan Road, Haidian District, Beijing 100083, P. R. China; Key Laboratory for Biomechanics and Mechanobiology of Ministry of Education, Beijing Advanced Innovation Centre for Biomedical Engineering, School of Engineering Medicine, School of Biological Science and Medical Engineering, Beihang University, No.37 Xueyuan Road, Haidian District, Beijing 100083, P. R. China; Key Laboratory for Biomechanics and Mechanobiology of Ministry of Education, Beijing Advanced Innovation Centre for Biomedical Engineering, School of Engineering Medicine, School of Biological Science and Medical Engineering, Beihang University, No.37 Xueyuan Road, Haidian District, Beijing 100083, P. R. China; Key Laboratory for Biomechanics and Mechanobiology of Ministry of Education, Beijing Advanced Innovation Centre for Biomedical Engineering, School of Engineering Medicine, School of Biological Science and Medical Engineering, Beihang University, No.37 Xueyuan Road, Haidian District, Beijing 100083, P. R. China; Key Laboratory for Biomechanics and Mechanobiology of Ministry of Education, Beijing Advanced Innovation Centre for Biomedical Engineering, School of Engineering Medicine, School of Biological Science and Medical Engineering, Beihang University, No.37 Xueyuan Road, Haidian District, Beijing 100083, P. R. China; Key Laboratory for Biomechanics and Mechanobiology of Ministry of Education, Beijing Advanced Innovation Centre for Biomedical Engineering, School of Engineering Medicine, School of Biological Science and Medical Engineering, Beihang University, No.37 Xueyuan Road, Haidian District, Beijing 100083, P. R. China; Key Laboratory for Biomechanics and Mechanobiology of Ministry of Education, Beijing Advanced Innovation Centre for Biomedical Engineering, School of Engineering Medicine, School of Biological Science and Medical Engineering, Beihang University, No.37 Xueyuan Road, Haidian District, Beijing 100083, P. R. China; Key Laboratory for Biomechanics and Mechanobiology of Ministry of Education, Beijing Advanced Innovation Centre for Biomedical Engineering, School of Engineering Medicine, School of Biological Science and Medical Engineering, Beihang University, No.37 Xueyuan Road, Haidian District, Beijing 100083, P. R. China; Department of Plant Genetics and Breeding, State Key Laboratory of Plant Physiology and Biochemistry & National Maize Improvement Center, China Agricultural University, No.17 Qinghua East Road, Haidian District, Beijing 100193, P. R. China; Key Laboratory for Biomechanics and Mechanobiology of Ministry of Education, Beijing Advanced Innovation Centre for Biomedical Engineering, School of Engineering Medicine, School of Biological Science and Medical Engineering, Beihang University, No.37 Xueyuan Road, Haidian District, Beijing 100083, P. R. China

## Abstract

**Abstract:**

Neoantigens are mutation-containing immunogenic peptides from tumor cells. Neoantigen intrinsic features are neoantigens’ sequence-associated features characterized by different amino acid descriptors and physical–chemical properties, which have a crucial function in prioritization of neoantigens with immunogenic potentials and predicting patients with better survival. Different intrinsic features might have functions to varying degrees in evaluating neoantigens’ potentials of immunogenicity. Identification and comparison of intrinsic features among neoantigens are particularly important for developing neoantigen-based personalized immunotherapy. However, there is still no public repository to host the intrinsic features of neoantigens. Therefore, we developed GNIFdb, a glioma neoantigen intrinsic feature database specifically designed for hosting, exploring and visualizing neoantigen and intrinsic features. The database provides a comprehensive repository of computationally predicted Human leukocyte antigen class I (HLA-I) restricted neoantigens and their intrinsic features; a systematic annotation of neoantigens including sequence, neoantigen-associated mutation, gene expression, glioma prognosis, HLA-I subtype and binding affinity between neoantigens and HLA-I; and a genome browser to visualize them in an interactive manner. It represents a valuable resource for the neoantigen research community and is publicly available at http://www.oncoimmunobank.cn/index.php.

**Database URL:**

http://www.oncoimmunobank.cn/index.php

**Key Points**

- GNIFdb contained comprehensive neoantigen intrinsic features for glioma and other tumors.
- Intrinsic features in GNIFdb can be exploited to prioritize neoantigens with immunogenic potential.
- HLA-I restricted neoantigen peptide sequence, neoantigen intrinsic features, neoantigen associated mutation, gene expression, HLA-I subtype and binding affinity between neoantigens and HLA-I were integrated in GNIFdb.
- A freely available and full functional website was built to search, browse and download all data in GNIFdb.

## Introduction

Nonsynonymous coding mutations may generate immunogenic peptides, neoantigens, that are presented to CD8+ T cells on restricted Human leukocyte antigen class I (HLA-I) subtype in selected tumor types such as melanoma (1), lung cancer (2), colorectal tumors (3) and Isocitrate Dehydrogenase (IDH) wild-type glioblastoma (GBM) (4). Neoantigens play pivotal roles in personalized immunotherapies, promoting tumor-specific T-cell responses and affecting antitumor immune responses in a number of preclinical models (5, 6). According to our previous study (4, 7, 8), it is found that neoantigen-based classifiers stratifies GBM patients with more favorable clinical outcome, but tumor neoantigen burden from a quantitative model fails to predict survival of GBM patients, suggesting that the underlying the intrinsic features of neoantigens may be distinct in patients with different prognosis. The intrinsic features refer to the neoantigens’ sequence-associated features characterized by amino Acid descriptors and physical–chemical properties. Therefore, studying intrinsic features may extend the knowledge of immunogenicity of neoantigens in different subtypes of glioma.

Glioma is a type of brain tumor consisting of multiple subtypes with few benefits achieved despite extensive treatment regiments during the last two decades (9–12). The tumor microenvironment dominated by mostly blood-derived macrophages and resident microglia actively operating to exclude T lymphocytes and undermine their functions limits a productive anti-tumor immunity in glioma (13–17). As higher mutation load is associated with increased tumor aggressiveness (18), mutation-generated neoantigens as inducer of immunogenic responses in glioma lies in their quality but not quantity (4, 19). There are multiple ways of evaluating the neoantigen qualities, including binding affinity between neoantigens and their corresponding restricted HLA-I subtypes, neoantigen intrinsic features and the expression levels of genes harboring the mutations generating neoantigens (4). Over the past years, several neoantigen-related databases (20–23) have been developed for cancers. However, none of these databases were designed to support glioma studies by integrating neoantigen intrinsic features in different glioma subtypes. Specifically, dbPepNeo (21) and NEPdb (24) focused on neoantigens manually curated from experimentally supported human tumor neoantigens. Immune Epitope Database (IEDB) (22), a gateway to global immune epitope information, was designed for general research purposes, mainly providing experimentally validated information of immune epitopes. TSNAdb (23) deposits neoantigens predicted by NetMHCpan based on somatic mutations of The Cancer Genome Atlas (TCGA) tumor samples and their restricted HLA subtypes in The Cancer Immunome Atlas (TCIA) (20). There is also a great need in glioma to build a specialized database that contains comprehensive neoantigen intrinsic features in all pathology, molecular genetics and epigenetics-based glioma classification subtypes with the aim to exploit the full potential of neoantigen intrinsic features for better evaluating the immunogenicity of neoantigen candidates.

Here, we develop GNIFdb (http://www.oncoimmunobank.cn/index.php), a database of HLA-I restricted neoantigen intrinsic features that integrates the genome-wide neoantigens of glioma covering all 20 subtypes according to up-to-date pathology, molecular genetics and epigenetics-based classification. GNIFdb also contains neoantigen intrinsic features for other four cancer types including lung cancer, melanoma, bladder cancer, and head and neck squamous cell carcinoma. Unlike existing databases, GNIFdb first includes intrinsic features of neoantigens from multiple sources and incorporates HLA-I restricted neoantigen peptide sequence, neoantigen intrinsic features, neoantigen-associated mutation, gene expression, HLA-I subtype and binding affinity between neoantigens and HLA-I. With these resources, this database facilitates systematic integrative investigation of immunogenicity of neoantigens in glioma and other cancer type and provides a configurable and interactive browser to visualize neoantigens as well as other related data.

## Materials and methods

### Data collection and preprocessing in glioma

Based on histological, molecular or both classifications, there are a total of 20 glioma subtypes, including GBM, astrocytoma, oligodendroglioma, oligoastrocytoma, co-deletion (1p and 19q), IDH mutant, IDH mutant co-deletion, IDH mutant non-co-deletion, G-CIMP high, G-CIMP low, IDH wild, classic, mesenchymal, neural, proneural, classic like, mesenchymal like, PA like, LGm6 GBM and primary GBM. In our previous study (4), we generated predicted neoantigens for the 20 glioma subtypes using whole exome sequencing data from TCGA with all the subtypes, mutation and survival information retrieved from the publication of our collaborator (Cohort 1) (25), and inferred neoantigens for IDH wild-type primary GBM from Asian population (Cohort 2) (4, 8). Specifically, missense mutation were used to generate all possible 9-mer peptides, with binding affinity of mutant and corresponding wild-type 9-mer peptides, relevant to the patient’s HLA-I alleles, predicted by netMHCpan-4.0. High- and low-affinity binders were defined as having IC_50_ equal or <500 nM or having IC_50_ >500 nM, respectively. Neoantigens were determined based on more stringent criteria, the mutant IC50 was <500 nM and IC50 of the corresponding wild-type binder, relevant to all HLA-I alleles of the patient, more than 500 nM (4). All neoantigens were categorized into different prognostic groups (<6 months, 6–12 months, 1–3 years, 3–5 years and >5 years) based on the survival of glioma patients. We also collected neoantigens of GBMs receiving anti-PD-1 immunotherapy treatment from our collaborator at Columbia University (Cohort 3) (26).

### Collection of neoantigens in other tumors

Additionally, we collected neoantigens publicly available in lung cancer, melanoma, bladder cancer and head and neck squamous cell carcinoma (Cohort 4) (27). Similarly, all neoantigens were categorized into different prognostic groups (<6 months, 6–12 months, 1–3 years, 3–5 years and >5 years) based on the survival of glioma patients. All genomic data were obtained from RefSeq and GenBank databases of NCBI and the UCSC genome browser.

### Collection of non-antigens

The non-antigens were determined by BLAST protein sequences randomly selected from Viral Bioinformatics Resource Center against hundreds of antigens we manually curated from the literature containing tested immunogenic protein data. A total of 48 210 9-mer peptides designated as false neoantigens were derived from 100 non-antigens by 9-mer sliding window with the step size of one amino acid.

### Calculation of neoantigen intrinsic features derived from AA descriptors

To calculate the neoantigen intrinsic features derived from Amino Acid (AA) descriptors, we used well-known methods including protFP (28, 29), blosum Indice (30), crucianiProperties (31), FASGAI (32), MS-WHIM (28, 29), kideraFactor (33), stScales (34), T-scale (32), zScales (35) and VHSE (36). The above neoantigens’ AA descriptors were computed based on the four conditions, respectively, including the complete sequence, the site of mutation along with each antigen and the dipeptides/tripeptides related to the mutation site, each absolute position along each antigen and related dipeptide/tripeptide composition, and the difference of each feature in the mutated versus reference antigen.

### Calculation of neoantigen intrinsic features derived from physical–chemical properties

To compute the intrinsic features derived from physical–chemical properties (37), R package ‘Peptides’(v2.4.2) was used to obtain features of auto-correlation, auto-covariance, Boman index, cross-covariance, hydrophobic moment, hydrophobicity, theoretical net charge, instability, and molecular weight. The above neoantigens’ physical–chemical properties were derived under the four conditions, respectively, including the complete sequence, the site of mutation along with each antigen and the dipeptides/tripeptides related to the mutation site, each absolute position along each antigen and related dipeptide/tripeptide composition, and the difference of each feature in the mutated versus reference antigen. In addition, we used the R command named ‘aaComp’ to retrieve features of Tiny, Small, Aliphatic, Aromatic, Non-polar, Polar, Basic, Acidic, which were derived based on whether the presence (1) or absence (0) of each feature under the same conditions.

### Calculation of neoantigen intrinsic features derived from Shannon entropy

Shannon entropy is an important index to measure the complexity at protein and residue levels. We calculated the Shannon entropy of a neoantigen using the following formula (37):

$${\rm{HS}} = - \mathop \sum \limits_{i = 1}^{20} {p_i}lo{g_2}{p_i}$$

 

$$H{R_i} = - {p_i}lo{g_2}{p_i}$$

where HS is Shannon entropy of a protein sequence and $H{R_i}$ is entropy of a residue type *i*. *p_i_* is the probability of the existence of a given amino acid in the sequence. We calculated the Shannon entropy of each neoantigen and its corresponding wild-type peptide. Cancer is characterized by the accumulation of mutations, so the analysis of mutant positions is valid. Therefore, the Shannon entropy of the dipeptides/tripeptides related to the mutation site and the entropy difference of mutations process were performed. The entropy of a residue type was also calculated for each neoantigen and its corresponding wild-type peptide.

### Calculation of neoantigen intrinsic features derived from mutations (AA properties)

The features describing overall content of mutant amino acid composition were also important. Based on mutation position and amino acid changes at mutation position, we calculated the intrinsic features derived from the number of times each amino acid appeared in the mutant peptide (noted as AA properties). Specifically, the AA property features were constructed in the way that the mutant amino acid demonstrating presence (1) or absence (0) of each amino acid type following, including the first or last three amino acid residues or middle residues of each neoantigen, the first or last amino acid residues of each neoantigen, the first or last two amino acid residues or middle residues of each neoantigen.

### Calculation of differential agretopicity index for neoantigens

Differential agretopicity index (DAI), which has been confirmed at a survival predictor in melanoma and non-small cell lung cancer (38), was proposed as a more accurate indicator of peptide immunogenicity (39, 40). We calculated DAI for each neoantigen by the difference in binding affinity between any neoantigen and its corresponding wild-type peptide.

### Summary of total data content

The current version of GNIFdb contains three independent glioma cohorts: Cohort 1 has 733 glioma patients including 20 pathological or molecular subtypes; Cohort 2 has 46 GBMs and Cohort 3 has 13 GBMs 2928 intrinsic features derived from amino acid descriptors, and physical–chemical properties were calculated for each neoantigen, resulting in 12 865 632 intrinsic features of 4394 neoantigens in three glioma cohorts combined (4091, 206 and 97 neoantigens for Cohorts 1, 2 and 3, respectively). GNIFdb also provides intrinsic features derived from neoantigens publicly available for four solid tumors in Cohort 4, including lung cancer (2619 neoantigens), melanoma (21 108 neoantigens), bladder cancer (1250 neoantigens) and head and neck squamous cell carcinoma (313 neoantigens) (Table 1). The intrinsic features of 48 210 9-mer peptides from non-antigens are incorporated in GNIFdb. Additionally, the binding affinity between each neoantigens and the corresponding restricted HLA was deposited in GNIFdb. DAI score was calculated and included in GNIFdb for all neoantigens of glioma cohorts and other tumor cohorts.

**Table 1.** GNIFdb data content and statistics

Cohort	Subtype	Neoantigens	AA features			Number of samples	
			AA descriptors	AA properties	Phys–chem prop	Expr data	Neoantigen data
Glioma (Cohort 1)	Glioma	4091	8 910 198	122 880	2 413 690	662	733
	Gbm	2223	4 841 694	355 680	1 311 570	152	285
	Astr	768	1 672 704	122 880	453 120	167	148
	Oligo	569	1 239 282	91 040	335 710	171	145
	Oligoastr	342	744 876	54 720	201 780	114	97
	1p19q Co-del	519	1 130 382	83 040	306 210	172	149
	IDHmut	1392	3 031 776	222 720	821 280	422	379
	IDHmut Co-del	515	1 121 670	82 400	303 850	168	147
	IDHmut Non-codel	878	1 912 284	140 480	518 020	254	232
	G-CIMP High	752	1 637 856	120 320	443 680	232	210
	G-CIMP Low	114	248 292	18 240	67 260	17	18
	IDHwt	2649	5 769 522	423 840	1 562 910	228	346
	Classic	913	1 988 514	146 080	538 670	85	112
	Mesenchymal	834	1 816 452	133 440	492 060	96	116
	Neural	460	1 001 880	73 600	271 400	110	97
	Proneural	1049	2 284 722	167 840	618 910	236	230
	Classic Like	973	2 119 194	155 680	574 070	68	120
	Mesenchymal Like	1134	2 469 852	181 440	669 060	98	156
	PA Like	129	280 962	20 640	76 110	25	14
	LGm6 Like	121	263 538	19 360	71 390	11	17
	IDHwt PriGbm	2132	4 643 496	341 120	1 257 880	135	262
Glioma (Cohort 2)	IDHwt PriGbm	206	448 668	32 960	121 540	–	46
Glioma (Cohort 3)	Gbm	97	211 266	15 520	57 230	–	12
Other tumors (Cohort 4)	Lung Cancer	2619	5 704 182	419 040	1 545 210	–	57
	Melanoma	21 108	45 973 224	3 377 280	12 453 720	–	151
	Bladder	1250	2 722 500	200 000	737 500	–	27
	HNSCC	313	681 714	50 080	184 670	–	12

Gbm: glioblastoma, Astr: astrocytoma, Oligo: oligodendrocyte, Oligoastr: oligoastrocytoma, IDHmut: IDH mutant, IDHmut Co-del: IDH mutant, 1p19q co-deletion, IDHmut non-codel: IDH mutant, 1p19q non-co-deletion, IDHwt: IDH wild-type, IDHwt PriGBM: IDH wild-type primary glioblastoma.

### Web interface implementation

**Figure 1.**
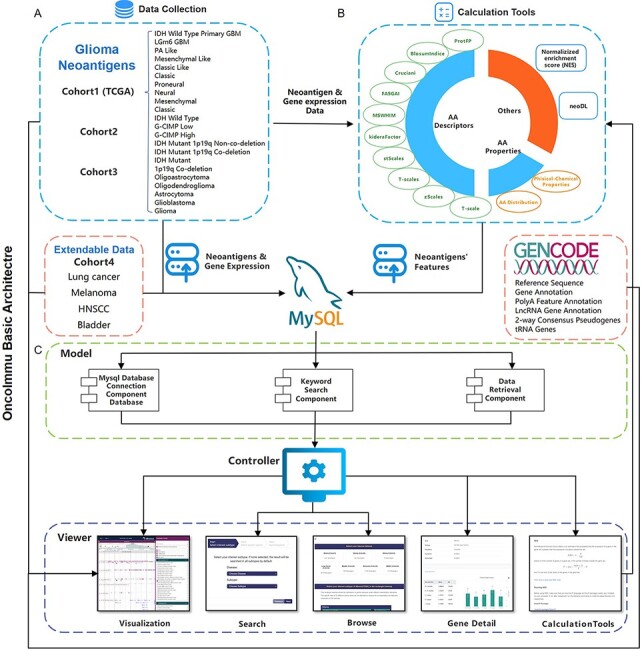
Schematic overview of data collection, data processing and key functionality of GNIFdb. (A) GNIFdb collects neoantigens from three independent glioma cohorts including 20 pathological or molecular subtypes in Cohort 1 from TCGA, 46 GBMs in Cohort 2 and 13 GBMs in Cohort 3. Cohort 4 includes four other solid tumors (lung cancer, melanoma, HNSCC and bladder cancer) as extendable data. (B) Intrinsic features of neoantigens in all the four cohorts were calculated using amino acid descriptors of protFP (28, 29), blosum Indice (30), crucianiProperties (31), FASGAI (32), MS-WHIM (28, 29), kideraFactor (33), stScales (34), T-scale (32), zScales (35), and VHSE (36), physical-chemical properties (37) of Tiny, Small, Aliphatic, Aromatic, Non-polar, Polar, Basic, Acidic, auto-correlation, auto-covariance, Boman index, crosscovariance, hydrophobic moment, hydrophobicity, theoretical net charge, instability, and molecular weight and AA properties. NES method and neoDL we previously developed are also included. (C) We implemented the GNIFdb following the Model-View-Controller architecture. The Model component handles data derived from multiple sources in MySQL databases and flat experimental data files, calculating the intrinsic features of neoantigens, performing statistical analysis and generating visualization plots. The View component provides heterogeneous and synchronized views to present the information and interact with the users. The front-end template engine of Bootstrap combined with HTML and JavaScript provide better visibility and usability of our functionality. The Controller component, a mediator between the Model and View components, deals with the application logic.

GNIFdb has been implemented with the use of MySQL (http://www.mysql.org), a free relational database management system, PHP, a popular general-purpose scripting language especially suited to web development, and Apache2 (http://httpd.apache.org/) on an Ubuntu Linux Server, following the Model-View-Controller architecture with the Model and View components being independent and loosely coupled for parallel development and simplification of updating and integrating new databases (Figure 1). Thus, GNIFdb is of good scalability, flexibility and extensibility. The Model component handles data derived from multiple sources from MySQL databases and flat experimental data files, which is the core functionality of the system including retrieving the intrinsic features of neoantigens, performing statistical analysis and generating visualization plots. The View component provides heterogeneous and synchronized views to present the information and interact with the users, which is the primary user interface component. The front-end template engine of Bootstrap combined with HTML and JavaScript provide great visibility and usability of our functionality, therefore enhancing browsing and searching abilities. The Controller component, a mediator between the Model and View components, deals with the application logic, which is tightly coupled with the independent components. GNIFdb is freely available at http://www.oncoimmunobank.cn/index.php.

## Results

### User-friendly browsing

**Figure 2.**
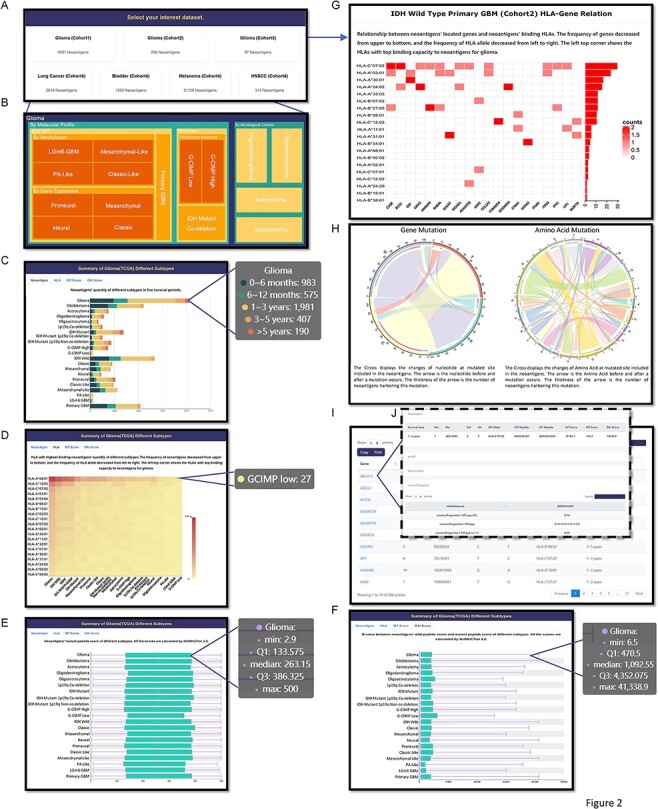
Browse page in GNIFdb showing information of neoantigens and their intrinsic features. (A) All cohorts. (B) The glioma subtypes in Cohort 1. (C) Neoantigens’ quantity of different glioma subtypes with five survival periods in Cohort 1. (D) The frequency of neoantigens and their corresponding HLA-I subtypes in different glioma subtypes of Cohort 1. (E) The distribution of neoantigens’ binding affinity score with their corresponding HLA-I subtypes in different glioma subtypes of Cohort 1. (F) The distribution of neoantigens’ DAI score in different glioma subtypes of Cohort 1. (G) The relationship between genes generating neoantigens and the HLA-I interacting with neoantigens. (H) The distribution of nucleotide or amino acid changes at mutated site in the neoantigens. (I) The list of genes harboring neoantigens, associated mutation site, the corresponding HLA-I subtypes interacting with the neoantigens and the survival time periods. (J) The intrinsic features of neoantigens associated with each gene.

GNIFdb collects 12 865 632 neoantigen intrinsic features and dedicates to store, browser and visualize intrinsic features derived from amino acid descriptors and physical–chemical properties of neoantigens in 20 glioma subtypes. GNIFdb also contains intrinsic features derived from neoantigens publicly available in lung cancer, melanoma, bladder cancer and head and neck squamous cell carcinoma and 9-mer peptides from non-antigens. The home page provides general information about GNIFdb. Interactive images displayed on browse page (http://www.oncoimmunobank.cn/item/browse), which provided quick links to access neoantigen intrinsic feature data, neoantigen peptide sequence, neoantigen-associated mutation information, gene expression, human leukocyte antigen (HLA) and HLA-binding affinity (Figure 2). Specifically, by clicking Cohort 1 (Figure 2A), users will be guided to select the glioma subtype they may have interests in (Figure 2B). Moving their cursor further down to ‘Neoantigen’ in ‘Summary of Glioma (TCGA) Different Subtypes’, the users can also examine the neoantigen quantity distribution among different glioma subtypes and compare the number of neoantigens among different survival subgroups in a specified glioma subtype (Figure 2C). By clicking ‘HLA’, users can review the number of neoantigens, the corresponding HLA-I subtypes and the number of patients with the selected HLA-I subtype among different glioma subtypes (Figure 2D). Users can also check the binding affinity score between neoantigens and their corresponding HLA subtypes among glioma subtypes by selecting ‘MT Score’ (Figure 2E). Similarly, DAI score distribution of neoantigens in different glioma subtypes can be shown in ‘DAI Score (Figure 2F). After selecting a cohort (glioma subtype for Cohort 1), users can access the relationship between genes harboring neoantigens and corresponding HLA-I subtypes in the selected glioma subtype (Figure 2G). They can also review the nucleotide or amino acid changes among mutations generating neoantigens (Figure 2H). By clicking a gene symbol, users can retrieve detailed neoantigen and associated intrinsic feature information (Figure 2I).

### Visualization page

**Figure 3.**
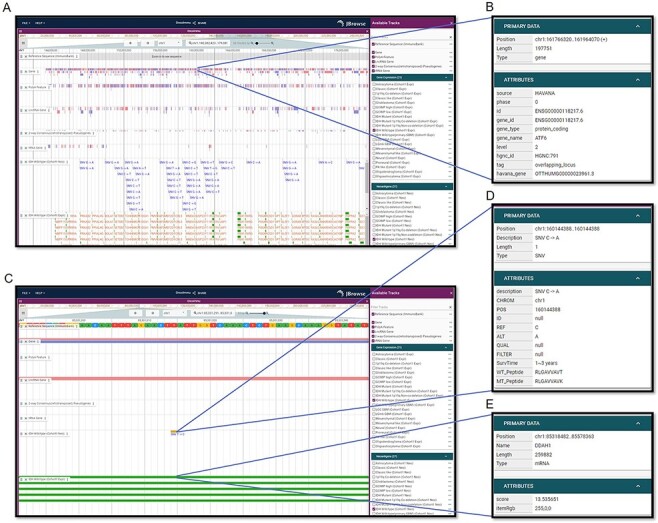
Visualization interfaces in GNIFdb. (A) Overview of the tracks corresponding to gene, mutation and neoantigens in a specified glioma subtype (IDH wild-type, Cohort 1 in this example). (B) The gene information includes gene symbol, gene length, location and gene type. (C) Zoomed-in tracks of mutation and gene expression shows the details of mutations and corresponding neoantigens (D) and gene expression (E).

To visualize neoantigens and associated information, GNIFdb deploys an interactive and user-friendly neoantigen browser built on JBrowse (41) (Figure 3). For each glioma subtype, the neoantigen browser has a variety of data tracks including Reference Sequence, Gene Annotation, PolyA Feature Annotation, LncRNA Gene Annotation, 2-wa Consensus (retrotransposed) Pseudogenes (predicted by the Yale and UCSC pipelines), tRNA Genes Predicted by tRNAscan-SE, Gene Expression and Neoantigens. Users are allowed to choose tracks of their interests and to zoom and scroll any region along the genome. The neoantigen browser is of great usefulness to investigate neoantigens of specific genes or regions across different glioma subtypes by taking account of multiple relevant data tracks (Figure 3A). For example, ATF6 regulates the expression of several pro-oncogenic proteins such as GRP78 and Notch1 and plays important roles in tumor growth and resistance to radiotherapy in GBM (42). If a user wants to examine the information associated with ATF6, by selecting ‘ATF6’ on the gene track, all detailed information of ATF6 will be displayed (Figure 3B), as well as for the information on other tracks (Figure 3A). Additionally, when a SNV is selected on the ‘Neoantigen’ track, the corresponding detailed information will be displayed including the amino acid sequence of neoantigen, the mutation generating this neoantigen and the survival of the patient having this mutation (Figure 3C and D). Similarly, the detailed information of the gene will be displayed as it is selected on the ‘Gene expression’ track (Figure 3C and E). Therefore, it is of high utility to investigate neoantigens of glioma subtype within specific genes or regions.

### Advanced search

**Figure 4.**
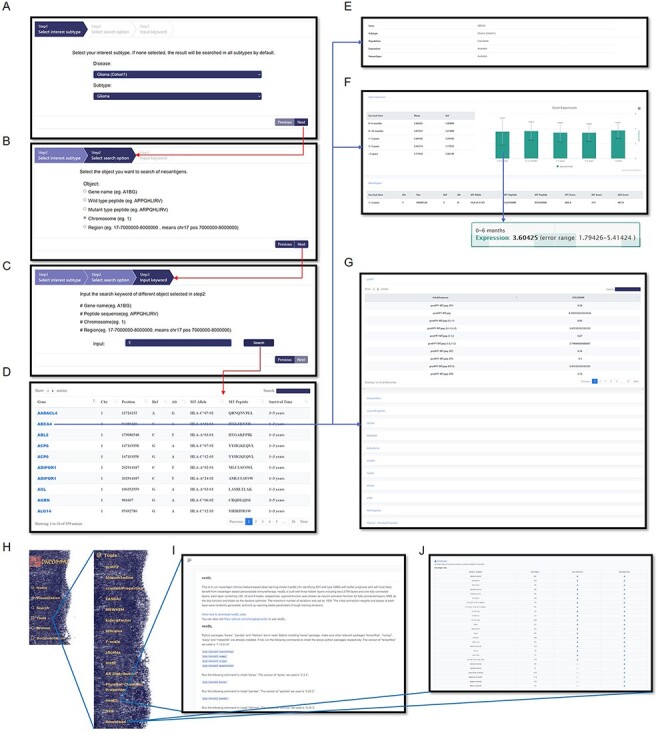
Information query and tools in GNIFdb. An example demonstrates that querying glioma (Cohort 1) (A), chromosome category (B) and chromosome 1 (C) generates the list of information associated gene, mutation, neoantigen, corresponding HLA-I subtype and survival time period (D) in the genomic region of interests. The query results also provide the summary of information availability (E) and detailed gene expression (F) and the full list of neoantigen intrinsic features (G). (H) Tools provide codes for calculating neoantigen intrinsic feature calculations, neoDL (I) and downloading of neoantigen and gene expression data for all cohorts (J).

To support information search and exploration, GNIFdb provides user-friendly web interfaces to search a diversity of information for a specific gene or genomic region in step-by-step manner (Figure 4). In addition to gene name searching, GNIFdb also supports searching by neoantigen peptide sequence, chromosome and the region associated with neoantigens. By specifying a disease and subtype in step 1 (Figure 4A), selecting gene name option at step 2 (Figure 4B) and inputting a chromosome number at step 3 (Figure 4C), users can obtain its mutation location, mutant and reference nucleotide, restricted HLA, neoantigens and survival time of the patient harboring this mutation (Figure 4D). By selecting the gene symbol, users can access to full information associated with this gene (Figure 4E), including the expression levels of this gene among patients with specified disease subtype (Figure 4F), all neoantigens detected in this gene and associated 12 major intrinsic feature categories (protFP, biosumIndice, crucianiProperties, FASGAI, MSWHIM, kideraFactor, stScales, tScales, zScales, VHSE, AA Properties and Physical-Chemical Properties) covering 2928 intrinsic feature types (Figure 4G).

### Intrinsic feature calculation tools

To help advanced users investigate their own neoantigens, GNIFdb provides ‘Tools’ that can be downloaded to calculate the intrinsic features of neoantigens. There are a total of 12 tools for calculating the neoantigen intrinsic features in the drop-down menus, including protFP, blosumIndice, crucianiProperties, FASGAI, MSWHIM, kideraFactor, stScales, T-scale, zScales, VHSE, AA Distribution and Physical-Chemical properties (Figure 4H). We also provide neoDL (a novel neoantigen intrinsic feature-based deep learning model) developed by our team (43) to download (Figure 4I). neoDL was built with three hidden layers including two LSTM layers and one fully connected layer, with each layer containing 128, 32 and 8 nodes, respectively. Sigmoid function was chosen as neuron activation function for fully connected layers, mean square error as the loss function and Adam as the iterative optimizer. Users can apply the neoDL to identify patients with better prognosis who will most likely benefit from neoantigen-based personalized immunotherapy. In addition, we also provide a tool named NES (normalized enrichment score) (4) for download, which is used to estimate the probability that the expression of a gene in the gene set is greater than the expression of a gene outside this set. In addition, each tool page has detailed manual. Users can optionally download these tools for their own good.

### Data download and help

GNIFdb provides a download page in Tools to quickly retrieve neoantigen and gene expression data of the desired glioma subtypes and other solid tumors. The download page shows the list of glioma subtypes and tumor types in a table manner (Figure 4J). For each glioma subtype or tumor type, two links are provided to download the entire data as a CSV file. The help page of GNIFdb in Documentation contains an extensive and detailed manual to aid new users in understanding the intrinsic features of neoantigens and layout of the website. Various sections of the page describe each feature of GNIFdb in detail and also provide information on how to use them.

### The utility of GNIFdb

Neoantigen intrinsic features, characterized by different amino acid descriptors and physical–chemical properties, play crucial roles in prioritizing neoantigens with potential immunogenicity and predicting patients with better survival. GNIFdb features integration and visualization of neoantigen intrinsic features as well as other related data, enabling identification of neoantigens with immunogenicity in different glioma subtypes and accordingly providing an important resource for development of neoantigen-based personalized immunotherapy. The utility of GNIFdb is further highlighted in the following use cases

### Case study 1: good prognostic GBMs having neoantigens with protective intrinsic features

Typically, GBM has a low mutation load (44, 45) and immunologically cold tumor microenvironment (46). The popular models including both neoantigen quantity and DAI model (difference between binding affinity of wild-type and mutant-type peptides) failed to predict the overall survival of IDH wild-type GBMs and 16 different glioma subgroups (4, 43). However, our previous study has shown a preferential enrichment of protective intrinsic features in IDH wild-type GBMs with the longest survival characterized by development and cell cycle associated with Gene Ontology pathways (43). This preference can also be observed in other glioma subtypes including GBM, Classical-like, Mesenchymal-like and Classical. Additionally, IDH wild-type GBMs in Asian population were also found having enrichment of protective intrinsic features. Twelve intrinsic features in categories of the molecular weight and molecular size/volume of the position 3,4 composed-dipeptide, and molecular electrostatic of the position 2–4 composed-tripeptide were protective factors in both TCGA cohort and GBMs in Asian. These prognostic intrinsic features of the neonatigens can be manipulated to identify neoantigens with high potential of immunogenicity.

### Case study 2: neoantigen intrinsic features-based deep learning model predicting good prognostic GBMs

We have previously observed the enrichment of protective intrinsic features in IDH wild-type GBMs with good overall survival (43). Currently, the vast majority of deep learning models (such as DeepLearning Model (47) and PASNet (48)) are based on gene expression, clinical information and medical image data, without direct help in finding patients who may benefit from neoantigen-based personalized immunotherapy. To identify GBMs with good prognosis and enriched protective intrinsic features, we constructed an intrinsic feature-based deep learning model including three hidden layers of two LSTM layers and one fully connected layer with 128, 32 and 8 nodes, respectively. The model was demonstrated to successfully stratify IDH wild-type GBMs into two subgroups with significantly different survival in two independent cohorts including TCGA cohort and a cohort of Asian population, even in some other high-grade glioma subtypes (43). The intrinsic feature-based deep learning model can be therapeutically exploited to identify IDH wild-type GBM with good prognosis who will most likely benefit from neoantigen-based personalized immunotherapy.

## Discussion

Different from extant databases, GNIFdb features (i) integrating neoantigen intrinsic features of full glioma subtypes including pathology and molecular-based classification; (ii) storing large amounts of neoantigens and mutations identified in glioma; (iii) interconnecting multiple related omics data and building a neoantigen browser for visualization of all types of data in a genomic context and (iv) allowing the online query of neoantigen intrinsic features, neoantigens and expression profiles for a given region or gene. Taken together, GNIFdb integrates and visualizes neoantigen intrinsic features as well as gene expression profiles and survival category, enabling identification of potential neoantigens in different glioma subtypes and providing an important resource for developing immune therapies.

GNIFdb is committed to integrating neoantigens and their intrinsic features in different glioma subtypes. Therefore, future developments for GNIFdb include incorporation of neoantigens and their intrinsic features from different tumors. Accordingly, GNIFdb will continue to integrate related types of data including expression profiles and SNP from different resources and add more neoantigen-related analysis tools. Considering the increasing number of neoantigens and their intrinsic features, it is also important to develop web pages and tools to allow the easy incorporation of new data. Furthermore, GNIFdb will also provide new possible intrinsic feature measurements in different cancer types and develop web interfaces to facilitate cross-cancer comparison of neoantigens and their intrinsic features in different cancer subtypes. The neoantigen browser will be further improved to support interactive visualization of big neoantigen data as well as other related data. In addition to the neoantigen and neoantigen intrinsic features generated by our team, we also invite the scientific community to submit their neoantigen data to GNIFdb and to build collaborations in improving the functionalities of GNIFdb.

## Data Availability

GNIFdb is available at http://www.oncoimmunobank.cn/item/browse to all users without any login or registration restrictions. The custom codes used for calculating neoantigen intrinsic features can be accessed at Tools page of GNIFdb or github https://github.com/zhangjbig/GNIFdb. All data can be downloaded from GNIFdb Tools/Download page.
